# Bilateral pulmonary artery banding for transposition of the great arteries complex with coarctation

**DOI:** 10.1093/icvts/ivac207

**Published:** 2022-08-05

**Authors:** Koji Miwa, Shigemitsu Iwai, Toshiaki Nagashima

**Affiliations:** Department of Cardiovascular Surgery, Osaka women’s and Children’s Hospital, Osaka, Japan; Department of Cardiovascular Surgery, Osaka women’s and Children’s Hospital, Osaka, Japan; Department of Cardiovascular Surgery, Osaka women’s and Children’s Hospital, Osaka, Japan

**Keywords:** Bilateral pulmonary artery banding, Transposition of great arteries, Coarctation of the aorta

## Abstract

Surgical approaches for transposition of the great arteries with aortic arch obstruction include primary repair and two-stage repair. However, neither approach provides a satisfactory outcome. We report a case of patient who underwent two-stage repair, wherein arterial switch operation combined with aortic arch reconstruction was preceded by bilateral pulmonary artery banding; this yielded good outcomes. This approach safely avoids primary repair in the neonatal period and allows for the opportunity to evaluate right ventricle outlet tract stenosis before the definitive repair.

## INTRODUCTION

The incidence of transposition of the great arteries (TGA) with aortic arch obstruction (AAO) reaches 50% in cases of Taussig–Bing anomaly; however, it is relatively rare (5%) in non-Taussig–Bing anomalies. Neonatal mortality is about 25–50% for TGA with AAO, respectively [1]. Surgical approaches to TGA with AAO include primary repair [arterial switch operation (ASO) plus aortic arch reconstruction (AAR)] and two-stage repair (ASO preceded by AAR and main pulmonary artery banding (mPAB)]. Despite reports of the superiority of the primary repair over the two-stage repair, its mortality and morbidity rates remain high [2, 3].

We report a case of a two-stage repair wherein ASO and AAR were preceded by bilateral pulmonary artery banding (bPAB).

## CASE REPORT

**Figure 1:**
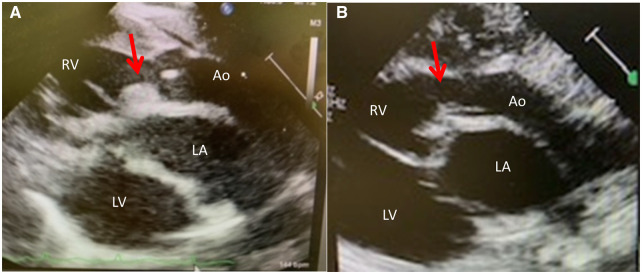
Echocardiography. (**A**) Preoperative: long-axis view showing right ventricular outflow tract stenosis. (**B**) Before definitive repair: long-axis view showing intact right ventricular outflow tract.

**Figure 2:**
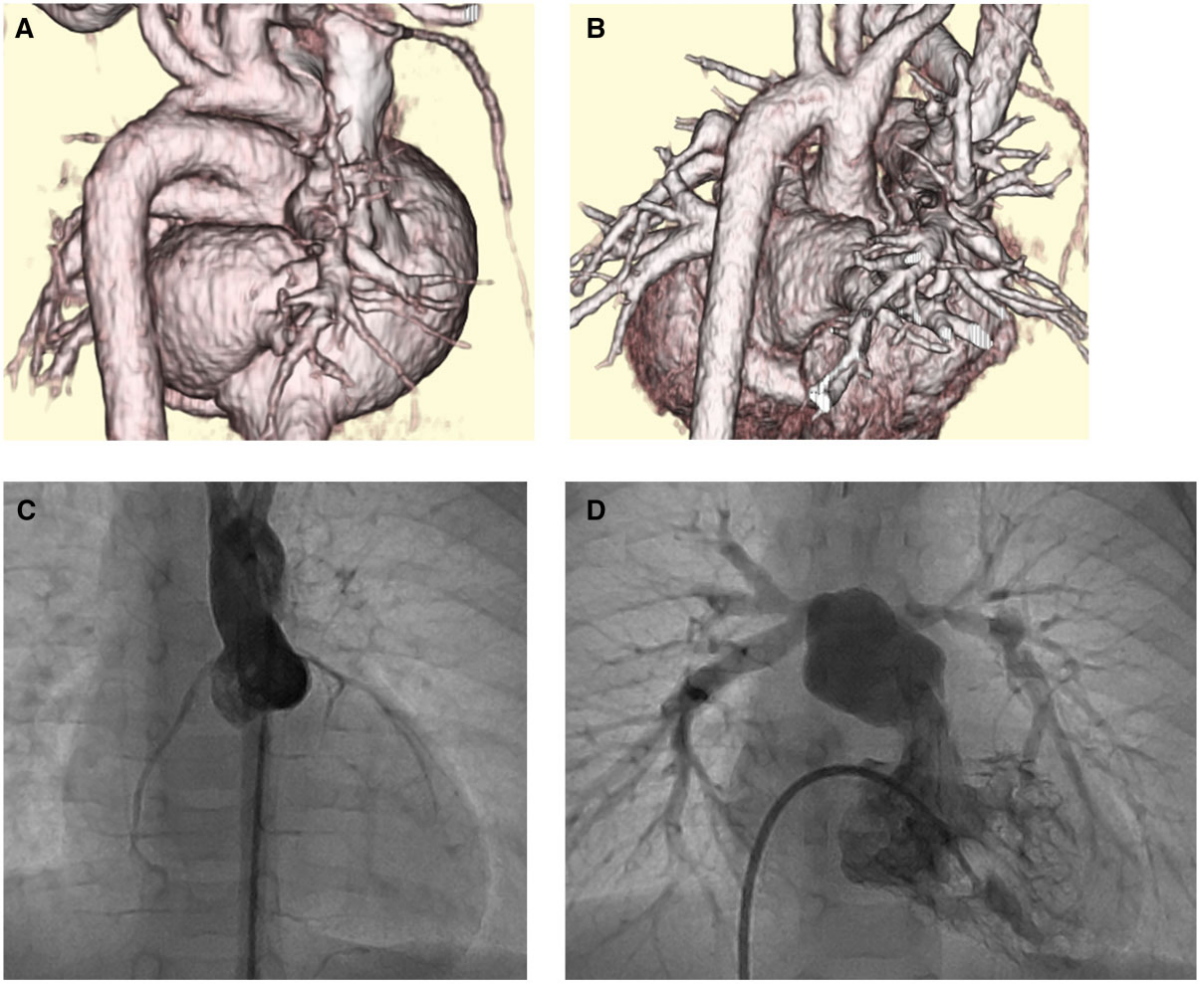
Computed tomography and cardiac catheterization. (**A**) Preoperative: coarctation of the aorta with hypoplastic aortic arch. (**B**) Postoperative: no re-coarctation of the aorta. (**C**) Postoperative aortic angiography shows intact coronary artery ostium and no aortic valve regurgitation. (**D**) Postoperative right ventriculography shows intact right ventricular outflow tract.

The patient was a male infant born at 41 gestational weeks (birthweight, 2976 g). Pre-ductal/post-ductal oxygen saturations were 88%/93% at birth. A postnatal echocardiography (Fig. 1A, Video 1) revealed TGA with perimembrenous type ventricular septal defect (VSD), coarctation of the aorta (CoA), right ventricular outflow tract stenosis (RVOTS), and coronary artery type of 1LCx-2R. The RVOTS measured 4.4 mm (72% of normal, *Z* score: −3.6). The cardiac computed tomography (Fig. 2A) revealed CoA with a hypoplastic aortic arch. Balloon atrioseptostomy was performed at birth to improve atrial level mixing. Thereafter, bPAB was performed 7 days postnatally, with a 0.4-mm polytetrafluoroethylene patch applied as banding tape. The patch was cut longitudinally to create a 2-mm-wide strip. The right and left pulmonary arteries were individually banded with an 11-mm tape. Pre-ductal/post-ductal oxygen saturations were 80%/85% after banding. The systemic blood pressure increased by 10 mmHg and venous oxygen saturation also increased from 40% to 56%. Postoperative echocardiography revealed a flow velocity of >3.5 m/s at both banding positions. Until definitive repair, we maintained the ductus arteriosus with prostaglandin E1. The postoperative course was uneventful, and the patient consistently gained weight. Echocardiography before the definitive repair (Fig. 1B, Video 2) showed spontaneous improvement in the RVOTS. At the age 1 month, we performed an ASO, combined with AAR and VSD closure. The postoperative course was uneventful, and ischaemic events or re-CoA, as visualized on cardiac computed tomography at 6 months (Fig. 2B), did not recur. Postoperative cardiac catheterization at 6 months (Fig. 2C and D) revealed an intact coronary artery ostium and right ventricle outlet tract.

## DISCUSSION

Our patient underwent a two-stage repair. However, due to concerns of RVOTS preoperatively, bPAB was performed instead of mPAB with AAR, as mPAB may increase the afterload and cause circulatory failure in both ventricles.

Choi *et al.* [3] reported reintervention for postoperative RVOTS progression following definitive repair and emphasized the importance of considering RVOTS preoperatively. However, despite the morphological suspicion of RVOTS being present preoperatively in patients with large VSDs, blood flow shunts from the right ventricle to the left ventricle without any obvious acceleration in the RVOTS, making its accurate evaluation difficult. Therefore, two-stage repair with bPAB enables an accurate evaluation and optimal surgical management of RVOTS in such cases.

In addition, it is reported that for patients diagnosed with interrupted aortic arch and subaortic stenosis (SAS), SAS improved after bPAB. They believed that bPAB improved SAS by increasing the blood flow through SAS, and this led the left ventricular outflow tract growth [4]. We supposed that the same mechanism led to the disappearance of the RVOTS in our case.

We believe that this approach safely avoids primary repair in the neonatal period and allows for the opportunity of RVOTS evaluation before the definitive repair.

## ETHICAL STATEMENT

The patient’s mother provided informed consent for the publication of the case report.

**Conflict of interest:** none declared.

## Reviewer information

Interactive CardioVascular and Thoracic Surgery thanks Katarzyna Januszewska, Karthik Vaidyanathan Ramakrishnan and the other, anonymous reviewer(s) for their contribution to the peer review process of this article.

## References

[1] Yada I, Wada H, Fujita H; Committee of Science. Thoracic and cardiovascular surgery in Japan during 2002: annual report by the Japanese Association for Thoracic Surgery. Jpn J Thorac Cardiovasc Surg 2004;52:491–508. 10.1007/s11748-003-0016-5 14717431

[2] Mohammadi S, Serraf A, Belli E, Aupecle B, Capderou A, Lacour-Gayet F et al Left-sided lesions after anatomic repair of transposition of the great arteries, ventricular septal defect, and coarctation: surgical factors. J Thorac Cardiovasc Surg 2004;128:44–52. 15224020 10.1016/j.jtcvs.2004.01.040

[3] Choi KH, Sung SC, Kim H, Lee HD, Ban GH, Kim G et al Transposition complex with aortic arch obstruction: outcomes of one-stage repair over 10 years. Pediatr Cardiol 2016;37:160–6. 26358472 10.1007/s00246-015-1258-6 PMC4737791

[4] Hirano Y, Inamura N, Kawazu Y, Aoki H, Kayatani F, Iwai S et al Evaluation of factors associated with achievement of biventricular repair after bilateral pulmonary artery banding in patients with interrupted aortic arch. World J Pediatr Congenit Heart Surg 2018;9:54–9. 29310563 10.1177/2150135117737685

